# Capacity, motivation, and opportunity model-derived taxonomy of pharmacist-led interventions applicable to people self-managing cancer medication

**DOI:** 10.3389/fpubh.2026.1772293

**Published:** 2026-03-09

**Authors:** Joana Ribeiro, Ramón Morillo-Verdugo, Filipa Alves da Costa

**Affiliations:** 1 Public Health and Medicines Use, Research Institute for Medicines (iMED), Faculty of Pharmacy, University of Lisbon, Lisbon, Portugal; 2 Department of Pharmacy Clinical Management, Valme University Hospital, Seville, Spain

**Keywords:** pharmacy service, hospital, ambulatory care, cancer, evidence-based pharmacy practice, taxonomy

## Abstract

**Introduction:**

With health systems facing increasing challenges, it is important to define new care models that may release some of the burden on the workforce, whilst maintaining quality and improving patient convenience. The objective of this study was to create a taxonomy of pharmacist-led interventions aimed at supporting improved health outcomes of people self-managing cancer medication.

**Methods:**

The list was developed following a literature search conducted in Cochrane Library and MEDLINE (PubMed) databases. The Capacity, Motivation and Opportunity (CMO) model developed for people living with HIV was used as theoretical framework to organise the pharmacist-led interventions emerging from literature. A panel of experts (hospital pharmacists with experience in oncology) selected through national hospital pharmacy societies was invited to participate in a consensusseeking Delphi survey, focusing on the most important and feasible interventions.

**Results:**

A total of 28 experts answered and rated 39 interventions. Whilst 35 of the proposed pharmacist-led interventions were considered important (median score above 8, on a scale of 1 to 9, without disagreement among experts), only eight were considered feasible for implementation in practice. The most frequently mentioned reasons for others not to be feasible were understaffing and excessive workload. Nevertheless, there were interventions considered vital for patients’ care and hence kept in the final taxonomy.

**Discussion:**

Future hospital-based studies should incorporate this taxonomy based on the CMO model to measure pharmacists’ interventions in real clinical practice.

## 1 Introduction

Health systems have been striving to progress to a person-centred approach, instead of the traditional and paternalistic disease-centred approach. In this context, pharmacists, as healthcare providers, have also progressed from a drug-oriented role, where drug distribution and production prevailed, to person-oriented services, where pharmaceutical care plays a crucial role (1).

Pharmaceutical care may be defined as a professional activity through which the pharmacist assumes responsibility for meeting patients’ care needs. Pharmacists plan strategies to align and achieve pharmacotherapy short- and medium-term goals and use available communication channels to continuously interact with the patient, so that close monitoring may contribute to improved health outcomes (2). Clinical pharmacists are healthcare professionals who provide this care to patients, possessing in-depth knowledge of medications, combined with a clinical and behavioural understanding (3).

Improved clinical and behavioural health outcomes rely on several factors, namely the person’s motivation and engagement with treatment, as well as self-management capability. The Capacity-Motivation- Opportunity (CMO) Pharmaceutical Care model is based on the three essential pillars orienting its name. Capacity refers to the provision of Pharmaceutical Care to a person, considering individual needs. Motivation concerns the establishment of tailored short, medium and long-term goals, in collaboration with the multidisciplinary health team, planning the necessary actions and interventions to meet the therapeutic goals. Opportunity is gained by close interaction with the person receiving care, being available when needed, responding to care needs in real and useful time, and resorting to new technologies whenever appropriate (4).

The initial CMO model was conceptualised by hospital pharmacists from the Spanish Society of Hospital Pharmacy for use in settings where people living with HIV are being treated obtaining positive outcomes (5, 6). Previous work from our group also involved developing a taxonomy to describe pharmacist-led interventions in accordance with the CMO-Pharmaceutical Care Model. This taxonomy was developed in several stages, including a literature review followed by a Delphi consensus panel aiming to select the most relevant interventions. The first stage led to the selection of 20 HIV-focused pharmacist-led interventions, among which medication review and prescription validation, as well as interventions fostering medication safety and adherence were considered key interventions and of prime concern. Additionally, care coordination and motivation-enhancing interventions were considered particularly impactful (7).

Although the CMO model has been primarily applied to people living with chronic HIV infection, it has been adapted in Spain to address the needs of people living with other chronic conditions, including people living with cancer (8–10). While stratification tools (11) have also been adapted for this setting, the taxonomy of cancer-focused pharmaceutical interventions has not yet been developed. The main objective of this study is to develop a taxonomy of pharmacist-led interventions aimed at supporting improved health outcomes of people self-managing cancer medication, based on the CMO Pharmaceutical Care model.

## 2 Materials and methods

The study was performed in two major phases:

1. A literature search to identify pharmacist-led interventions aimed at supporting improved health outcomes among people self-managing cancer medication. The process was guided by a structured search strategy using predefined MeSH terms, restricted to studies published between April 2012 and April 2022
2. Consensus seeking.

- The development of a literature-extracted list of pharmacist-led interventions to construct a survey to be used in a consensus development technique to identify the most relevant interventions. For each intervention, a classification was created so that experts involved could consider the two main domains thought to influence implementation in practice: clinical relevance and practical feasibility, both rated using an ordinal scale of 1 (clearly not necessary) to 9 (clearly necessary) points.
- A consensus development two-phase Delphi technique for achieving the cancer-centered taxonomy. Pharmaceutical societies from Spain and Portugal identified experts in oncology, aiming to include 50% of experts from each country. Consensus cut-off value was set at 7 and 9 without discordance.

The study was ethically approved by the Ethics Committee of the Portuguese Institute of Oncology in Lisbon (protocol code: UIC/1510, date: 14 July 2022).

### 2.1 Literature review

The objective of this phase was to extract a list of pharmacist-led interventions to support people living with cancer treated with medications they can self-manage, and to develop a classification proposal to be submitted to the expert panel. A Preferred Reporting Items for Systematic reviews and Meta-Analyses extension for Scoping Reviews (PRISMA-ScR) approach was followed to guide the literature search (12). In parallel, an electronic brainstorming exercise was conducted to guide the entire literature review process (13). It consisted of questions and proposals exchanged via email among members of the research team. The outputs from brainstorming sessions were the search equation, inclusion or exclusion of certain articles, grouping text portions extracted from literature, and positioning of interventions within the pillars of the CMO model.

The search was conducted in the Cochrane Library and in MEDLINE (PubMed) database through a search equation inspired by the PICO method (see Equation S1). The inclusion of studies from the Cochrane Library ensured that the search captured interventions from other databases and from the grey literature. Furthermore, an analysis across all Cochrane Review Groups demonstrated that clinical and pharmaceutical interventions had greater coverage in PubMed than other health interventions (14).

During full-text revision, the pharmacist-led interventions were extracted into a data table (Microsoft Excel®) that included a description of the study objectives, methods, and main results. Studies were included if they involved any pharmacist-led intervention aimed at improving health outcomes, were carried out in outpatients with cancer, and were primary (case report, case series, case–control study, cohort study, clinical trial) or secondary (systematic or non-systematic review, meta-analysis) studies employing quantitative or qualitative methodology, and observational or experimental designs, either cross-sectional or longitudinal (prospective or retrospective). Editorials were not considered.

Studies were excluded if professionals conducting the intervention were not identified, if they were carried out in community pharmacies (because the CMO model was specifically designed for outpatient hospital pharmacy consultations), or if they focused on pharmacovigilance, pharmacoeconomic, pharmacokinetic, pharmacodynamic, and pharmacogenomic outcomes, as most of these do not necessarily require direct patient contact, hence falling outside the scope of our study. The list of pharmacist-led interventions was then categorized under the Capacity, Motivation and Opportunity pillars according to the definitions established in the CMO model.

### 2.2 Consensus seeking

The objective of this phase was to reach expert consensus on a standardized classification of pharmacist-led interventions that support improved health outcomes in people self-managing cancer medication, using the Delphi-RAND/UCLA Appropriateness Method and standardized reporting (DELPHISTAR) (15, 16). Collaborative agreements were signed with the Portuguese Association of Hospital Pharmacists (APFH) and the Spanish Society of Hospital Pharmacy (SEFH) to support oncology expert identification. A panel of 28 hospital pharmacists was selected, 14 from Spain and 14 from Portugal.

After signing the informed consent, experts individually assessed the importance and feasibility of each pharmacist-led intervention within the corresponding CMO pillar and provided observations or suggestions to improve the classification (*first Delphi round*). Experts were blinded to each other’s responses to ensure anonymity throughout all phases of the study. The importance of a pharmacist-led intervention was defined as its potential impact on improving health outcomes in people self-managing cancer medication. The feasibility of a pharmacist-led intervention was defined as the likelihood that a pharmacist could perform the intervention under routine working conditions.

Responses were compiled and analyzed by the research team. Interventions were classified according to the degree of agreement among experts, based on the following criteria:

- *Adequate:* group median between 7 and 9 without disagreement;
- *Uncertain:* group median between 4 and 6 or any median with disagreement;
- *Inadequate:* group median between 1 and 3 without disagreement.

Interventions considered adequate were immediately retrieved. Each expert subsequently received a personalized report containing the group median for uncertain and inadequate interventions, along with their own initial responses. This new assessment form enabled each expert to re-evaluate the importance and feasibility of those interventions (*second Delphi round*). This iterative process minimized external influences, ensuring that final scores reflected the independent judgment of each expert.

Finally, interventions were reclassified by the research team as adequate, uncertain or inadequate. Those rated as adequate for both importance and feasibility, either in the first or second round, were included in the final taxonomy of pharmacist-led interventions.

A post-hoc sub-analysis was conducted to explore potential differences in outcomes across countries. Scores from the first Delphi round were stratified by country and analyzed using the predefined criteria described above, enabling the identification of interventions achieving consensus in none, one, or both domains (relevance and feasibility) for Spain, for Portugal, or for both countries. An identical procedure was subsequently applied to the data obtained from the second Delphi round.

## 3 Results

### 3.1 Literature review

**Figure 1:**
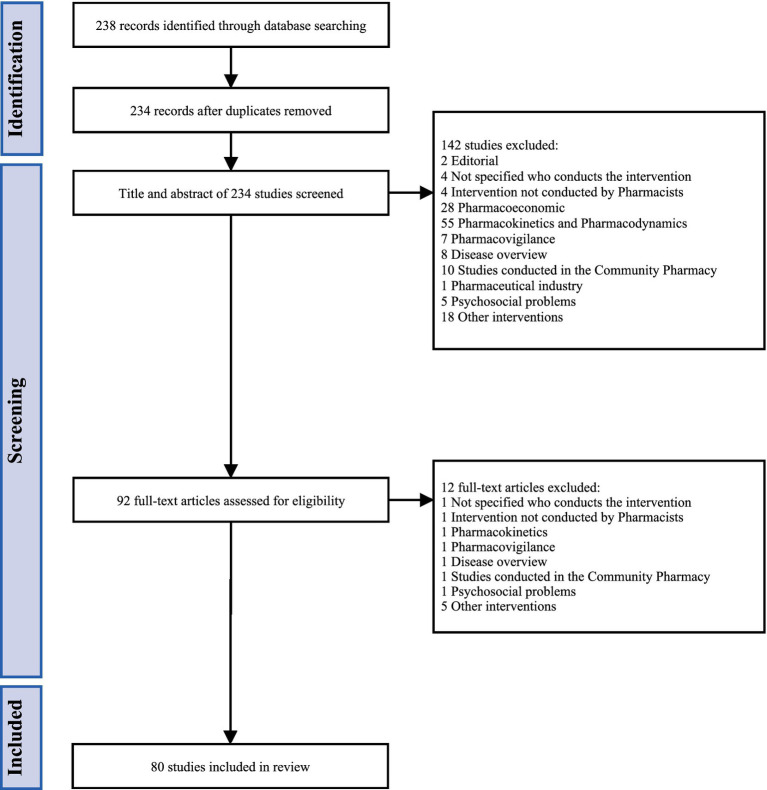
PRISMA diagram representing the process for identification of studies enabling extraction of cancer-focused pharmacist-led interventions.

The literature search retrieved a total of 238 articles (see Figure 1). After removing duplicates, 234 studies were obtained, of which 92 were selected for further evaluation. Of these, 80 were included for extraction of pharmacist-led interventions aimed at supporting improved health outcomes in people self-managing cancer medication. A total of 233 portions of text were extracted *ipsis verbis* from the selected studies, which, following duplicate removal, resulted in 186 unique interventions. These portions were inductively analyzed to group pharmacist-led interventions with similar definitions based on descriptions made in the respective full texts. This process resulted in a final list of 44 distinct pharmacist-led interventions.

**Figure 2:**
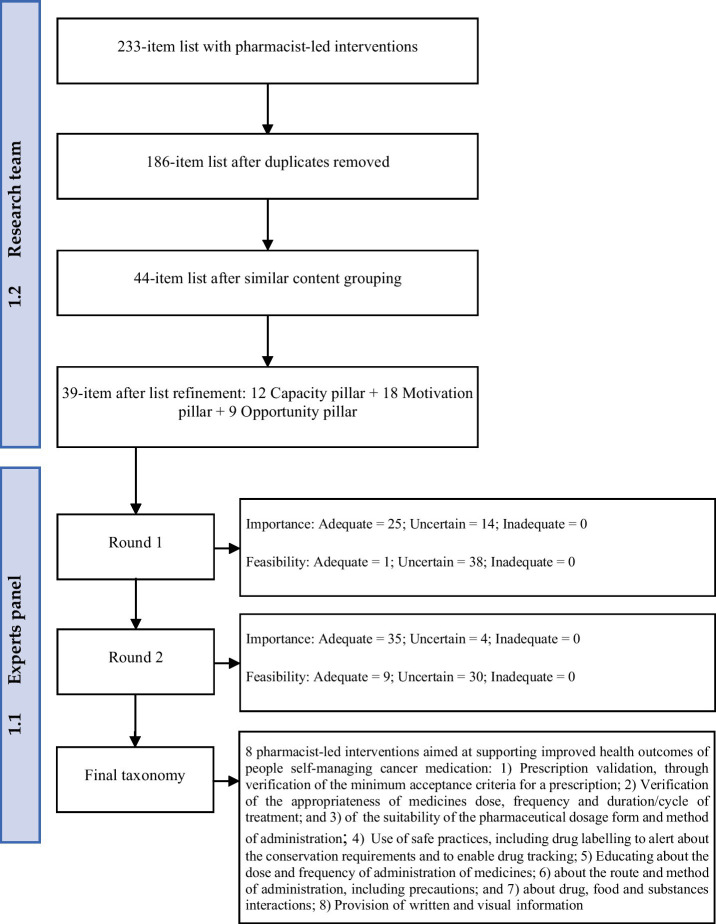
Flow diagram to demonstrate the development of a taxonomy from an initial list of pharmacist-led interventions aiming to support improved health outcomes of people self-managing cancer medication.

The research team held regular discussions to review and validate the selection process, leading to the identification of additional equivalent interventions, resulting in a final list of 39 interventions, each of which was assigned to one of the three CMO pillars—Capacity, Motivation or Opportunity—based on team consensus (see Figure 2 and Supplementary Table S1).

**Figure 3:**
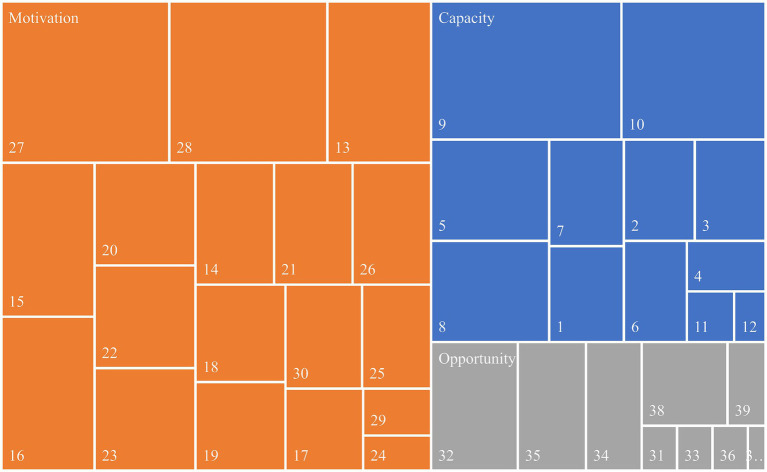
Proportional representation of pharmacist-led interventions identified in the reviewed literature, per CMO pillar (the description of the listed pharmacist-led interventions is presented in Supplementary Table S1).

Literature mainly highlights pharmacist-led interventions within the Motivation pillar (see Figure 3). These involve monitoring and managing medication adherence and addressing adverse effects. Another key focus was on patient education and counselling, including in written, particularly about medication intake. In the oncology context, warnings about storing, handling, and disposing of medications were considered crucial.

In the Capacity pillar, the focus was on reviewing and optimising therapy and discussing any therapy changes with a multidisciplinary team. Within this pillar the importance of medicines reconciliation and verifying the appropriateness of dosage was also highlighted. Lastly, interventions in the Opportunity pillar were those least reported in literature. The most frequently mentioned and considered relevant within this pillar was the clarification of drug-related questions by pharmacists.

### 3.2 Consensus seeking

The characteristics of healthcare professionals selected through national hospital pharmacy societies to participate in the study are presented in Table 1. The sample was intentionally heterogeneous in professional experience to allow for the opportunity to gain different perspectives. Half the participants were working at university hospitals, and most had clinical roles.

**Table 1:** Healthcare professionals’ characteristics in relation to their professional experience, role, and hospital type.

Variable	Category	*n*	%
Professional experience	1–5 years	6	21%
	6–10 years	3	11%
	11–15 years	5	18%
	16–20 years	6	21%
	> 21 years	8	29%
Pharmacist role	Clinical	19	68%
	Management	9	32%
Type of hospital	University hospital	14	50%
	Cancer centre	6	21%
	General hospital	8	29%

The first Delphi round achieved a response rate of 79% (*n* = 22), and the second round achieved a response rate of 75% (n = 21).

The expert panel classified 25 of the 39 pharmacist-led interventions as adequate in the first round, and 10 more in the second round, totalling 35 interventions considered adequate, while four remained of uncertain importance.

Only one of the 39 pharmacist-led interventions was rated as feasible in the first round (Verification of the appropriateness of medicines dose, frequency, and duration or cycle of treatment), whereas seven more interventions were considered feasible in the second round. The remaining 31 interventions were classified as uncertain in terms of feasibility. These eight pharmacist-led interventions based on the CMO model, considered both important and feasible, were included in the final taxonomy (Table 2).

**Table 2:** Taxonomy of cancer-focused pharmacist-led interventions based on the CMO model.

CMO pillar	Pharmacist-led interventions
Capacity	Prescription validation, through verification of the minimum acceptance criteria for a prescription (2)
	Verification of the appropriateness of medicines dose, frequency, and duration/cycle of treatment (5)
	Verification of the suitability of the pharmaceutical dosage form and method of administration (6)
	Use of safe practices, including drug labelling to alert about the conservation requirements and to enable drug tracking (12)
Motivation	Educating about the dose and frequency of administration of medicines (15)
	Educating about the route and method of administration, including precautions (16)
	Educating about drug, food and substance interactions (18)
	Provision of written and visual information (22)

Substances interactions include alcohol, tobacco, herbal substances and illegal drugs.

**Figure 4:**
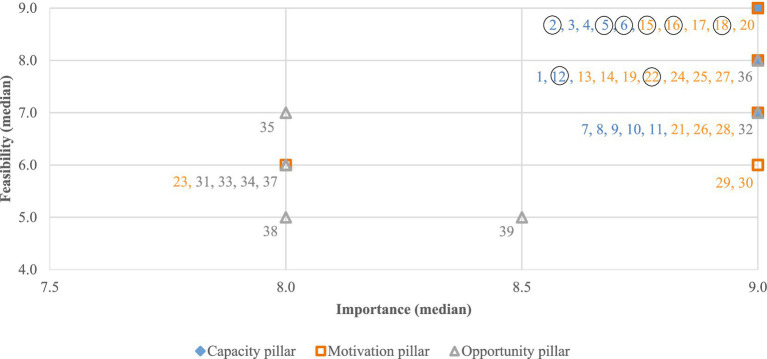
Results of the second Delphi round: Importance-Feasibility matrix of Pharmacist-led interventions. The eight interventions enclosed in a circle were included in the final taxonomy (the description of the listed pharmacist-led interventions is presented in Supplementary Table S1; the eight retained pharmacist-led interventions marked with circles are detailed in Table 2).

Within the Capacity pillar, the interventions considered of uncertain feasibility had median scores between 7 and 9, but with disagreement among experts. In contrast, in the Motivation and Opportunity pillars, nine items had median scores of 5 or 6, as shown in Figure 4 and Supplementary Table S2.

The observations written by experts about pharmacist-led interventions in the Opportunity pillar suggest that these interventions are still poorly established in daily practice and dependent on several factors such as the hospital resources or the patient’s digital literacy.

Accordingly, of all pharmacist-led interventions aimed at improving health outcomes in people self-managing cancer medication presented to the expert panel (derived from the literature), eight interventions were consensually rated as both important and feasible in current daily practice across two Delphi rounds (see Figure 2).

A post-hoc sub-analysis of the data obtained in each country identified differences mainly in the degree of agreement between experts, on the level of practical feasibility of pharmacist-led interventions (see Supplementary Table S3), suggesting 10 additional interventions would have been in the final taxonomy if only Spanish experts were considered. These were:

- Medicines selection and implementation of treatment protocols in collaboration with the multidisciplinary team (1);
- Validation of medicine (or protocol) indication and diagnosis (3);
- Verification of medicine (or protocol) contraindications and performance status, comorbidities, allergies and pregnancy (4);
- Medication review and optimization of therapeutic regimen (identification and analysis of drug-related problems [e.g., drug interactions, potentially inappropriate medications, undesirable effects, adherence or administration issues, medication omission and duplication, medication ineffectiveness]) (9);
- Motivational interviewing and patient counseling (13);
- Information on the starting date and planned duration of treatment (17);
- Teaching for prevention, healthy lifestyle and self-care (e.g., diet, fluid intake, physical activity, pregnancy prevention) (19);
- Information on special precautions for storage, handling and disposal of medicinal products (20);
- Patient assessment and monitoring (24);
- Use of telepharmacy (35).

Among Portuguese experts, there were only three pharmacist-led interventions considered both important and feasible not retrieved in the Iberian taxonomy:

- Information about disease-related symptoms (e.g., pain) (21);
- Verifying patient’s understanding (25);
- Assessment, monitoring and management of undesirable effects (28).

## 4 Discussion

The study identified key pharmacist-led interventions for people self-managing cancer medication and assessed their importance and practical feasibility. Patient counselling, including education on medication use, and safe practices with antineoplastic agents were unanimously highlighted by experts and literature, positioning the pharmacist as a key element in ensuring patient safety (17).

Other interventions were also considered by experts as crucial and feasible, such as prescription validation (including dose, frequency, and treatment duration), as well as verification of pharmaceutical form suitability and routes of administration. These are consistent with the minimum applicable regulatory and legal requirements, which strongly influence the roles and responsibilities of pharmacists (18, 19). In our study we have asked participants to consider the current daily practice as an indicator of short-term feasibility and therefore the findings reflect current regulatory frameworks and scopes of practice, the latter being sometimes fostered by scientific and professional societies and going beyond what could be expected by blunt interpretation of the law.

Interventions such as monitoring of laboratory parameters, medicines reconciliation, and follow-up were consistently rated as important but frequently limited in practice, not having been included in the final taxonomy. Reasons identified by experts were staff shortages and high patient volumes, although literature shows that these interventions may be delivered by other healthcare professionals (20). Likewise, interventions involving multidisciplinary teamwork, such as medicines selection and protocol implementation, were considered essential but with restricted feasibility. The reasons identified were insufficient group meetings and oversaturation of communication channels. This corroborates existing evidence about multidisciplinary teams being gold standards yet facing implementation challenges in practice (21).

Telepharmacy and digital tools are underrepresented on the literature*, an*d therefore, this naturally impacts the developed taxonomy, where the domain of opportunity is underrepresented compared to the other two domains of the CMO model. Experts identified information technology (IT) infrastructure, reluctance to change, and patient digital literacy as major constraints. The literature consistently described these three factors, in addition to challenges in data protection and a lack of healthcare professionals’ training programmes (22, 23). In contrast, population-based studies focusing digital health estimated medium to high literacy levels in Spain and Portugal, which leads us to think that in the future this domain may grow as IT progresses and become embedded in pharmaceutical interventions (24, 25).

The aforementioned interventions, not included in the final taxonomy but unanimously considered important, are aligned with Hematology/Oncology Pharmacist Association (HOPA) best practices (26). Practical constraints reflect previous research on the adoption of the CMO model and broader health system limitations highlighted by Organisation for Economic Co-operation and Development (OECD) reports and systematic reviews (22, 27–29).

Furthermore, there seems to be a strong dependency of feasibility on local care pathways and existing organisational models. Post-hoc sub-analysis showed a higher degree of agreement among experts from the same country. These results may be linked to existing similarities in hospital pharmacy scope of practice within each country. As such, our findings suggest that the level of practice is currently more advanced in Spain, as 10 additional pharmacist-led interventions were considered feasible by Spanish experts. Another hypothesis is that the level of maturity in the implementation of the CMO model varies across countries, leading pharmacists in Spain to be more conscious about advanced or more recent pharmacist-led interventions, such as conducting motivational interviews or using telepharmacy.

The strengths of this study include the homogeneity of training and expertise of the participants involved, and the dispersion and representativeness of the hospitals where they work. Additionally, the methodological advantage of the Delphi process adopted, to ensure participants anonymity during rounds, likely reduced response bias. While the literature review was centred on the most widely used database, complemented with an evidence-based specific database for systematic reviews, other databases could have been sought, and this restriction may have led to the omission of other relevant oncology-focused pharmacist-led interventions. Other limitations include potential selection bias associated with the recruiting bodies and the limited generalizability of findings to local contexts. Expert agreement varied across interventions, particularly where practical feasibility was contingent on workload or system infrastructure.

The taxonomy developed allows for the consistent classification of oncology-focused pharmacist-led interventions delivered in the hospital setting. By providing a structured framework for recording and evaluating interventions, contrasting these against performance indicators may support benchmarking exercises for strategic decision-making. Indicators derived from the taxonomy can identify care gaps and relate these with desirable interventions to enhance clinical, economic, and organizational outcomes, including supporting their prioritization. A list of 39 pharmacist-led interventions aimed at supporting improved health outcomes of people self-managing cancer medication was identified, albeit only eight were considered crucial and feasible for implementation in the current structure and organization of the Iberian healthcare system. Our recommendations would be to promote the implementation of the eight-priority pharmacist-led interventions and subsequently and progressively invest in the remaining 27 by creating the legal, regulatory and organisation conditions that would facilitate feasibility, including by enhancing the workforce capacity and efficiency of workflows. Future hospital-based studies should incorporate this taxonomy based on the CMO model to measure pharmacists’ interventions in real clinical practice.

## Data Availability

The raw data supporting the conclusions of this article will be made available by the authors, without undue reservation.
