# Supplementary material for: Capacity, motivation, and opportunity model-derived taxonomy of pharmacist-led interventions applicable to people self-managing cancer medication
Source: Front Public Health. 2026 Mar 9;14:1772293. doi: 10.3389/fpubh.2026.1772293 (PMC13006579; doi:10.3389/fpubh.2026.1772293)
Supplement: Supplementary file 1 [file Data_Sheet_1.pdf]

# *Supplementary Material*

## 1 Supplementary Data

**Equation S1:** Search equation used in MEDLINE (PubMed) database.

((pharmac* care[Title/Abstract]) OR (pharmac* consultation[Title/Abstract]) OR (hospital pharmacy services[Title/Abstract]) OR (pharmac* services[Title/Abstract]) OR (pharmacist intervention*[Title/Abstract]) OR (pharmac*[Title/Abstract])) AND ((oral chemotherapy[Title/Abstract]) OR (oral antineoplastic agent[Title/Abstract]) OR (oral antineoplastic drug[Title/Abstract]) OR (oral antineoplastic medicine[Title/Abstract]) OR (oral antineoplastic treatment[Title/Abstract]) OR (oral anticancer agent[Title/Abstract]) OR (oral anticancer drug[Title/Abstract]) OR (oral anticancer medicine[Title/Abstract]) OR (oral anticancer treatment[Title/Abstract]) OR (oral oncolytic*[Title/Abstract]))

## 2 Supplementary Tables

**Table S1:** Final list of pharmacist-led interventions aimed at supporting improved health outcomes for cancer patients

| CMO pillar | Pharmacist-led interventions |
|---|---|
| Capacity | **1.** Medicines selection and implementation of treatment protocols in collaboration with the multidisciplinary team<br>**2.** Prescription validation through verification of the minimum acceptance criteria for a prescription<br>3. Validation of medicine (or protocol) indication and diagnosis<br>4. Verification of medicine (or protocol) contraindications and performance status, comorbidities, allergies and pregnancy<br>5. Verification of the appropriateness of medicines dose, frequency, and duration/cycle of treatment<br>6. Verification of the suitability of the pharmaceutical dosage form and method of administration<br>7. Adequacy of monitoring laboratory parameters and other tests (e.g., electrocardiogram)<br>**8.** Medicines reconciliation (obtaining the best possible medication history through multiple sources of information, identifying and resolving discrepancies)<br>**9.** Medication review and medicines optimisation (identification and analysis of drug-related problems [e.g., drug interactions, potentially inappropriate medications, undesirable effects, adherence or administration issues, medication omission and duplication, medication ineffectiveness])<br>**10.** Discussion of changes in therapy with the multidisciplinary team (e.g., medicine change, dose or pharmaceutical dosage form changes)<br>**11.** Planning and scheduling pharmaceutical consultations<br>**12.** Use of safe practices, including drug labelling to alert about the conservation requirements and to enable drug tracking |
| Motivation | **13.** Motivational interviewing and patient counselling<br>14. Educating about the therapeutic objectives<br>15. Educating about the dose and frequency of administration of medicines<br>16. Educating about the route and method of administration, including precautions<br>17. Educating about the initiation date and the planned duration of treatment<br>18. Educating about drug, food and substance interactions |

| | |
|---|---|
| | 19. Providing information about prevention, healthy living and self-care (e.g., diet, fluid intake, physical activity, pregnancy prevention)<br>20. Information on special precautions for storage, handling and disposal of medicinal products<br>21. Information about disease-related symptoms (e.g., pain)<br>22. Provision of written and visual information<br>23. Additional educational sessions (e.g., on adherence, side effects)<br>**24.** Patient assessment and monitoring<br>25. Checking patients' understanding of information provided<br>26. Monitoring of parameters according to patient and medicines characteristics (e.g., weight, blood pressure, kidney and liver function)<br>27. Assessment, monitoring, and management of medication adherence<br>28. Assessment, monitoring, and management of undesirable effects<br>29. Measurement of patient-reported outcomes and patient-reported experiences (PROMs and PREMs)<br>**30.** Development of therapeutic plans (plan the objectives and actions) in collaboration with the multidisciplinary team |
| Opportunity | **31.** Assessment of health literacy and the amount of information to provide<br>**32.** Clarification of medication-related doubts through direct communication channels between the hospital pharmacist and the patient (e.g., via telephone, text message, e-mail, mobile application, website)<br>**33.** Emotional support facilitation through available resources (e.g., patient associations, reliable sources of information)<br>**34.** Multidisciplinary and multidimensional follow-up (e.g., referral to social worker, nutritionist, psychologist, family physician, home support)<br>**35.** Use of telepharmacy<br>36. Medication dispensing and informed delivery to the patient<br>37. Patient interview via videoconference<br>38. Pharmacotherapeutic follow-up through new technologies<br>39. Coordination of care (e.g., coordination with community pharmacist) |

**Table S2:** Assessment of the importance and feasibility of pharmacist-led interventions aimed at improved health outcomes for cancer patients

| CMO Pillar | Pharmacist-led intervention number | Criteria I = Importance F = Feasibility | First round | | | Second round | | |
|---|---|---|---|---|---|---|---|---|
| | | | Median | Interquartile Range | Degree of agreement | Median | Interquartile Range | Degree of agreement |
| Capacity | 1. | I | 9 | 0,0 | Adequate | - | - | - |
| | | F | 8 | 2,0 | Uncertain | 8 | 0,8 | Uncertain |
| | 2. | I | 9 | 0,0 | Adequate | - | - | - |
| | | F | 9 | 0,0 | Uncertain | 9 | 0,0 | Adequate |
| | 3. | I | 9 | 0,0 | Adequate | - | - | - |
| | | F | 9 | 0,0 | Uncertain | 9 | 0,0 | Uncertain |
| | 4. | I | 9 | 0,0 | Adequate | - | - | - |
| | | F | 9 | 2,0 | Uncertain | 9 | 0,0 | Uncertain |
| | 5. | I | 9 | 0,0 | Adequate | - | - | - |
| | | F | 9 | 0,0 | Adequate | - | - | - |
| | 6. | I | 9 | 0,3 | Adequate | - | - | - |
| | | F | 9 | 2,0 | Uncertain | 9 | 0,0 | Adequate |
| | 7. | I | 9 | 1,3 | Uncertain | 9 | 0,0 | Adequate |
| | | F | 7 | 2,0 | Uncertain | 7 | 1,0 | Uncertain |
| | 8. | I | 9 | 0,3 | Uncertain | 9 | 0,0 | Adequate |
| | | F | 7 | 2,3 | Uncertain | 7 | 1,0 | Uncertain |
| | 9. | I | 9 | 0,0 | Adequate | - | - | - |
| | | F | 7 | 1,0 | Uncertain | 7 | 0,5 | Uncertain |

| | | | | | | | | |
|---|---|---|---|---|---|---|---|---|
| | 10. | I | 9 | 1,0 | Adequate | - | - | - |
| | | F | 7 | 1,3 | Uncertain | 7 | 1,0 | Uncertain |
| | 11. | I | 9 | 1,3 | Uncertain | 9 | 1,0 | Adequate |
| | | F | 7 | 2,5 | Uncertain | 7 | 1,0 | Uncertain |
| | 12. | I | 9 | 1,0 | Adequate | - | - | - |
| | | F | 8 | 2,0 | Uncertain | 8 | 1,0 | Adequate |
| **Motivation** | 13. | I | 9 | 1,0 | Adequate | - | - | - |
| | | F | 8 | 2,0 | Uncertain | 8 | 0,0 | Uncertain |
| | 14. | I | 9 | 1,0 | Adequate | - | - | - |
| | | F | 8 | 3,0 | Uncertain | 8 | 0,5 | Uncertain |
| | 15. | I | 9 | 0,0 | Adequate | - | - | - |
| | | F | 9 | 1,0 | Uncertain | 9 | 0,0 | Adequate |
| | 16. | I | 9 | 0,0 | Adequate | - | - | - |
| | | F | 9 | 0,3 | Uncertain | 9 | 0,0 | Adequate |
| | 17. | I | 9 | 1,0 | Uncertain | 9 | 0,5 | Uncertain |
| | | F | 9 | 1,0 | Uncertain | 9 | 0,0 | Adequate |
| | 18. | I | 9 | 0,0 | Adequate | - | - | - |
| | | F | 9 | 1,3 | Uncertain | 9 | 0,5 | Adequate |
| | 19. | I | 9 | 1,0 | Uncertain | 9 | 0,0 | Adequate |
| | | F | 8 | 3,0 | Uncertain | 8 | 0,5 | Uncertain |
| | 20. | I | 9 | 0,3 | Adequate | - | - | - |
| | | F | 9 | 0,3 | Uncertain | 9 | 0,0 | Uncertain |
| | 21. | I | 9 | 1,0 | Uncertain | 9 | 1,0 | Adequate |

| | | | | | | | | |
|---|---|---|---|---|---|---|---|---|
| | | F | 7 | 3,0 | Uncertain | 7 | 1,0 | Uncertain |
| | 22. | I | 9 | 0,0 | Adequate | - | - | - |
| | | F | 8 | 2,0 | Uncertain | 8 | 0,0 | Adequate |
| | 23. | I | 8 | 2,0 | Adequate | - | - | - |
| | | F | 6 | 2,0 | Uncertain | 6 | 1,0 | Uncertain |
| | 24. | I | 9 | 0,3 | Adequate | - | - | - |
| | | F | 8 | 2,3 | Uncertain | 8 | 0,8 | Uncertain |
| | 25. | I | 9 | 0,0 | Adequate | - | - | - |
| | | F | 8 | 2,0 | Uncertain | 8 | 0,0 | Uncertain |
| | 26. | I | 9 | 1,0 | Adequate | - | - | - |
| | | F | 7 | 1,3 | Uncertain | 7 | 1,0 | Uncertain |
| | 27. | I | 9 | 0,0 | Adequate | - | - | - |
| | | F | 8 | 2,3 | Uncertain | 8 | 1,0 | Uncertain |
| | 28. | I | 9 | 0,0 | Adequate | - | - | - |
| | | F | 7 | 1,5 | Uncertain | 7 | 0,8 | Uncertain |
| | 29. | I | 9 | 1,0 | Adequate | - | - | - |
| | | F | 6 | 2,0 | Uncertain | 6 | 0,5 | Uncertain |
| | 30. | I | 9 | 1,0 | Adequate | - | - | - |
| | | F | 6 | 1,0 | Uncertain | 6 | 0,5 | Uncertain |
| **Opportunity** | 31. | I | 8 | 0,0 | Uncertain | 8 | 0,0 | Adequate |
| | | F | 6 | 2,0 | Uncertain | 6 | 0,0 | Uncertain |
| | 32. | I | 9 | 1,0 | Uncertain | 9 | 0,0 | Adequate |
| | | F | 7 | 1,3 | Uncertain | 7 | 0,5 | Uncertain |
| | 33. | I | 8 | 1,3 | Uncertain | 8 | 0,5 | Uncertain |

| | | | | | | | | |
|---|---|---|---|---|---|---|---|---|
| | | F | 6 | 2,0 | Uncertain | 6 | 0,5 | Uncertain |
| | 34. | I | 8 | 1,0 | Uncertain | 8 | 1,0 | Adequate |
| | | F | 6 | 2,5 | Uncertain | 6 | 1,0 | Uncertain |
| | 35. | I | 8 | 1,0 | Uncertain | 8 | 1,0 | Adequate |
| | | F | 7 | 1,0 | Uncertain | 7 | 0,0 | Uncertain |
| | 36. | I | 9 | 0,5 | Uncertain | 9 | 0,0 | Uncertain |
| | | F | 8 | 2,0 | Uncertain | 8 | 1,5 | Uncertain |
| | 37. | I | 8 | 1,3 | Uncertain | 8 | 0,0 | Uncertain |
| | | F | 6 | 3,3 | Uncertain | 6 | 2,0 | Uncertain |
| | 38. | I | 9 | 2,0 | Uncertain | 8 | 1,0 | Adequate |
| | | F | 5 | 2,3 | Uncertain | 5 | 1,0 | Uncertain |
| | 39. | I | 9 | 1,3 | Adequate | - | - | - |
| | | F | 5 | 2,0 | Uncertain | 5 | 1,0 | Uncertain |

**Table S3:** Assessment of the importance and feasibility of pharmacist-led interventions by country (post-hoc sub-analysis).

| Spain | | | | | | | | |
|---|---|---|---|---|---|---|---|---|
| **CMO Pillar** | **Pharmacist-led intervention number** | **Criteria I = Importance F = Feasibility** | **First round** | | | **Second round** | | |
| | | | Median | Interquartile Range | Degree of agreement | Median | Interquartile Range | Degree of agreement |
| Capacity | 1. | I | 9,0 | 0,0 | Adequate | - | - | - |
| | | F | 9,0 | 2,0 | Uncertain | 8,0 | 0,0 | Adequate |
| | 2. | I | 9,0 | 0,0 | Adequate | - | - | - |
| | | F | 9,0 | 0,0 | Adequate | 9,0 | 0,0 | Adequate |
| | 3. | I | 9,0 | 0,0 | Adequate | - | - | - |
| | | F | 9,0 | 0,0 | Adequate | 9,0 | 0,5 | Uncertain |
| | 4. | I | 9,0 | 0,5 | Adequate | - | - | - |
| | | F | 9,0 | 1,5 | Uncertain | 9,0 | 0,0 | Adequate |
| | 5. | I | 9,0 | 0,0 | Adequate | - | - | - |
| | | F | 9,0 | 0,0 | Adequate | - | - | - |
| | 6. | I | 9,0 | 1,0 | Adequate | - | - | - |
| | | F | 9,0 | 2,0 | Uncertain | 9,0 | 0,5 | Adequate |
| | 7. | I | 8,0 | 2,0 | Uncertain | 9,0 | 0,0 | Adequate |
| | | F | 7,0 | 2,5 | Uncertain | 7,0 | 1,0 | Uncertain |
| | 8. | I | 9,0 | 1,0 | Adequate | 9,0 | 0,0 | Adequate |
| | | F | 7,0 | 3,0 | Uncertain | 7,0 | 1,0 | Uncertain |
| | 9. | I | 9,0 | 0,5 | Adequate | - | - | - |
| | | F | 7,0 | 1,5 | Uncertain | 7,0 | 0,0 | Adequate |
| | 10. | I | 9,0 | 1,0 | Adequate | - | - | - |
| | | F | 7,0 | 1,0 | Uncertain | 7,0 | 0,5 | Uncertain |
| | 11. | I | 8,0 | 2,0 | Uncertain | 9,0 | 1,0 | Adequate |
| | | F | 8,0 | 2,5 | Uncertain | 7,0 | 1,0 | Uncertain |
| | 12. | I | 9,0 | 0,5 | Adequate | - | - | - |
| | | F | 8,0 | 2,0 | Uncertain | 8,0 | 0,5 | Adequate |
| Motivation | 13. | I | 9,0 | 1,0 | Adequate | - | - | - |
| | | F | 8,0 | 2,5 | Uncertain | 8,0 | 0,0 | Adequate |
| | 14. | I | 9,0 | 1,0 | Adequate | - | - | - |
| | | F | 9,0 | 2,5 | Uncertain | 8,0 | 1,0 | Uncertain |
| | 15. | I | 9,0 | 0,0 | Adequate | - | - | - |
| | | F | 9,0 | 0,0 | Adequate | 9,0 | 0,0 | Adequate |
| | 16. | I | 9,0 | 0,0 | Adequate | - | - | - |
| | | F | 9,0 | 0,0 | Adequate | 9,0 | 0,0 | Adequate |
| | 17. | I | 9,0 | 1,5 | Adequate | 9,0 | 0,3 | Adequate |
| | | F | 9,0 | 0,0 | Adequate | 9,0 | 0,0 | Adequate |
| | 18. | I | 9,0 | 0,0 | Adequate | - | - | - |
| | | F | 9,0 | 2,0 | Uncertain | 9,0 | 1,0 | Adequate |

| | | | | | | | | |
|---|---|---|---|---|---|---|---|---|
| | 19. | I | 8,0 | 2,0 | Uncertain | 9,0 | 1,0 | Adequate |
| | | F | 8,0 | 2,0 | Uncertain | 8,0 | 0,0 | Adequate |
| | 20. | I | 9,0 | 0,5 | Adequate | - | - | - |
| | | F | 9,0 | 0,0 | Adequate | 9,0 | 0,0 | Uncertain |
| | 21. | I | 8,0 | 2,0 | Uncertain | 8,5 | 1,0 | Adequate |
| | | F | 7,0 | 3,0 | Uncertain | 7,0 | 1,0 | Uncertain |
| | 22. | I | 9,0 | 0,5 | Adequate | - | - | - |
| | | F | 9,0 | 1,5 | Adequate | 8,0 | 0,5 | Adequate |
| | 23. | I | 7,0 | 1,5 | Adequate | - | - | - |
| | | F | 6,0 | 2,0 | Uncertain | 6,0 | 0,5 | Uncertain |
| | 24. | I | 9,0 | 0,5 | Adequate | - | - | - |
| | | F | 8,0 | 2,5 | Uncertain | 8,0 | 0,5 | Adequate |
| | 25. | I | 9,0 | 0,0 | Adequate | - | - | - |
| | | F | 9,0 | 1,5 | Uncertain | 8,0 | 0,0 | Uncertain |
| | 26. | I | 9,0 | 1,0 | Adequate | - | - | - |
| | | F | 7,0 | 1,0 | Uncertain | 7,0 | 1,0 | Uncertain |
| | 27. | I | 9,0 | 0,0 | Adequate | - | - | - |
| | | F | 8,0 | 2,5 | Uncertain | 8,0 | 0,5 | Uncertain |
| | 28. | I | 9,0 | 0,5 | Adequate | - | - | - |
| | | F | 7,0 | 2,0 | Uncertain | 7,0 | 0,5 | Uncertain |
| | 29. | I | 9,0 | 1,0 | Adequate | - | - | - |
| | | F | 6,0 | 1,5 | Uncertain | 6,0 | 0,5 | Uncertain |
| | 30. | I | 9,0 | 1,0 | Adequate | - | - | - |
| | | F | 6,0 | 1,0 | Uncertain | 6,0 | 0,0 | Uncertain |
| **Opportunity** | 31. | I | 8,0 | 1,0 | Uncertain | 8,0 | 0,0 | Adequate |
| | | F | 6,5 | 2,5 | Uncertain | 6,0 | 0,5 | Uncertain |
| | 32. | I | 9,0 | 2,0 | Uncertain | 9,0 | 0,0 | Adequate |
| | | F | 7,0 | 2,0 | Uncertain | 7,0 | 1,5 | Uncertain |
| | 33. | I | 8,0 | 2,0 | Adequate | 8,0 | 0,0 | Adequate |
| | | F | 6,0 | 2,0 | Uncertain | 6,0 | 0,5 | Uncertain |
| | 34. | I | 8,0 | 2,0 | Uncertain | 8,0 | 1,0 | Adequate |
| | | F | 5,0 | 2,5 | Uncertain | 6,0 | 1,0 | Uncertain |
| | 35. | I | 8,0 | 2,0 | Uncertain | 8,0 | 1,0 | Adequate |
| | | F | 7,0 | 1,5 | Uncertain | 7,0 | 0,5 | Adequate |
| | 36. | I | 9,0 | 1,5 | Uncertain | 9,0 | 1,0 | Uncertain |
| | | F | 9,0 | 2,0 | Uncertain | 8,0 | 1,0 | Uncertain |
| | 37. | I | 8,0 | 2,0 | Uncertain | 8,0 | 0,0 | Uncertain |
| | | F | 7,0 | 2,5 | Uncertain | 7,0 | 2,0 | Uncertain |
| | 38. | I | 8,0 | 2,0 | Adequate | 8,0 | 1,3 | Adequate |
| | | F | 5,0 | 2,5 | Uncertain | 5,0 | 1,0 | Uncertain |
| | 39. | I | 8,0 | 2,0 | Adequate | - | - | - |
| | | F | 5,0 | 1,5 | Uncertain | 5,0 | 1,0 | Uncertain |

| Portugal | | | | | | | | |
|---|---|---|---|---|---|---|---|---|
| CMO Pillar | Pharmacist-led intervention number | Criteria I = Importance F = Feasibility | First round | | | Second round | | |
| | | | Median | Interquartile Range | Degree of agreement | Median | Interquartile Range | Degree of agreement |
| Capacity | 1. | I | 9,0 | 0,5 | Adequate | - | - | - |
| | | F | 8,0 | 2,0 | Uncertain | 8,0 | 1,0 | Uncertain |
| | 2. | I | 9,0 | 0,0 | Adequate | - | - | - |
| | | F | 9,0 | 0,3 | Adequate | 9,0 | 0,0 | Adequate |
| | 3. | I | 9,0 | 1,0 | Adequate | - | - | - |
| | | F | 9,0 | 2,0 | Uncertain | 9,0 | 0,0 | Adequate |
| | 4. | I | 9,0 | 0,0 | Adequate | - | - | - |
| | | F | 8,0 | 2,5 | Uncertain | 9,0 | 1,0 | Uncertain |
| | 5. | I | 9,0 | 0,5 | Adequate | - | - | - |
| | | F | 9,0 | 1,5 | Adequate | - | - | - |
| | 6. | I | 9,0 | 0,0 | Adequate | - | - | - |
| | | F | 9,0 | 1,5 | Adequate | 9,0 | 0,0 | Adequate |
| | 7. | I | 9,0 | 0,5 | Uncertain | 9,0 | 0,0 | Adequate |
| | | F | 7,0 | 1,5 | Uncertain | 7,0 | 1,0 | Uncertain |
| | 8. | I | 9,0 | 0,0 | Uncertain | 9,0 | 0,0 | Adequate |
| | | F | 6,0 | 3,0 | Uncertain | 7,5 | 2,0 | Uncertain |
| | 9. | I | 9,0 | 0,0 | Adequate | - | - | - |
| | | F | 7,0 | 1,5 | Uncertain | 7,0 | 1,0 | Uncertain |
| | 10. | I | 9,0 | 0,0 | Adequate | - | - | - |
| | | F | 8,0 | 2,5 | Uncertain | 8,0 | 1,5 | Uncertain |
| | 11. | I | 9,0 | 0,5 | Adequate | 9,0 | 0,0 | Adequate |
| | | F | 7,0 | 4,0 | Uncertain | 7,0 | 4,5 | Uncertain |
| | 12. | I | 9,0 | 1,0 | Adequate | - | - | - |
| | | F | 9,0 | 1,0 | Uncertain | 9,0 | 0,0 | Adequate |
| Motivation | 13. | I | 9,0 | 0,0 | Adequate | - | - | - |
| | | F | 7,0 | 6,3 | Uncertain | 8,0 | 1,0 | Uncertain |
| | 14. | I | 9,0 | 0,5 | Adequate | - | - | - |
| | | F | 8,0 | 4,5 | Uncertain | 8,0 | 0,8 | Uncertain |
| | 15. | I | 9,0 | 0,0 | Adequate | - | - | - |
| | | F | 9,0 | 1,0 | Uncertain | 9,0 | 0,0 | Adequate |
| | 16. | I | 9,0 | 0,0 | Adequate | - | - | - |
| | | F | 9,0 | 1,5 | Uncertain | 9,0 | 0,0 | Adequate |
| | 17. | I | 9,0 | 1,0 | Uncertain | 9,0 | 1,0 | Uncertain |
| | | F | 8,0 | 2,5 | Uncertain | 9,0 | 0,8 | Adequate |
| | 18. | I | 9,0 | 0,0 | Adequate | - | - | - |
| | | F | 9,0 | 1,0 | Uncertain | 9,0 | 0,0 | Adequate |
| | 19. | I | 9,0 | 1,0 | Adequate | 9,0 | 0,0 | Adequate |
| | | F | 8,0 | 4,0 | Uncertain | 8,0 | 1,0 | Uncertain |

| | | | | | | | | |
|---|---|---|---|---|---|---|---|---|
| | 20. | I | 9,0 | 0,5 | Adequate | - | - | - |
| | | F | 9,0 | 1,5 | Uncertain | 9,0 | 0,0 | Adequate |
| | 21. | I | 9,0 | 0,5 | Adequate | 9,0 | 0,3 | Adequate |
| | | F | 7,0 | 2,0 | Uncertain | 7,0 | 1,0 | Adequate |
| | 22. | I | 9,0 | 0,0 | Adequate | - | - | - |
| | | F | 7,0 | 2,0 | Uncertain | 8,0 | 1,0 | Adequate |
| | 23. | I | 8,0 | 1,0 | Adequate | - | - | - |
| | | F | 7,0 | 3,0 | Uncertain | 6,5 | 1,0 | Uncertain |
| | 24. | I | 9,0 | 0,5 | Adequate | - | - | - |
| | | F | 7,0 | 3,5 | Uncertain | 8,0 | 1,0 | Uncertain |
| | 25. | I | 9,0 | 0,5 | Adequate | - | - | - |
| | | F | 8,0 | 3,0 | Uncertain | 8,0 | 0,8 | Adequate |
| | 26. | I | 9,0 | 0,5 | Adequate | - | - | - |
| | | F | 7,0 | 1,0 | Uncertain | 7,0 | 1,0 | Uncertain |
| | 27. | I | 9,0 | 0,0 | Adequate | - | - | - |
| | | F | 8,0 | 2,0 | Uncertain | 8,0 | 1,0 | Uncertain |
| | 28. | I | 9,0 | 0,0 | Adequate | - | - | - |
| | | F | 7,5 | 1,8 | Uncertain | 7,0 | 1,0 | Adequate |
| | 29. | I | 9,0 | 1,0 | Adequate | - | - | - |
| | | F | 7,0 | 3,0 | Uncertain | 6,0 | 2,3 | Uncertain |
| | 30. | I | 9,0 | 0,8 | Adequate | - | - | - |
| | | F | 6,0 | 3,3 | Uncertain | 6,0 | 1,8 | Uncertain |
| **Opportunity** | 31. | I | 8,0 | 1,0 | Adequate | 8,5 | 1,0 | Adequate |
| | | F | 6,0 | 2,0 | Uncertain | 6,0 | 0,0 | Uncertain |
| | 32. | I | 9,0 | 0,5 | Adequate | 9,0 | 0,3 | Adequate |
| | | F | 7,0 | 1,0 | Uncertain | 7,0 | 0,8 | Uncertain |
| | 33. | I | 8,0 | 1,0 | Uncertain | 8,0 | 1,8 | Uncertain |
| | | F | 5,0 | 2,5 | Uncertain | 6,0 | 1,0 | Uncertain |
| | 34. | I | 9,0 | 1,0 | Adequate | 8,0 | 1,0 | Adequate |
| | | F | 6,0 | 3,5 | Uncertain | 6,0 | 1,8 | Uncertain |
| | 35. | I | 8,5 | 1,0 | Adequate | 8,0 | 1,0 | Adequate |
| | | F | 7,0 | 1,5 | Uncertain | 7,0 | 1,0 | Uncertain |
| | 36. | I | 9,0 | 0,0 | Adequate | 9,0 | 0,0 | Adequate |
| | | F | 7,5 | 2,8 | Uncertain | 8,0 | 2,0 | Uncertain |
| | 37. | I | 8,0 | 1,0 | Uncertain | 8,0 | 1,5 | Uncertain |
| | | F | 5,0 | 4,0 | Uncertain | 5,5 | 2,0 | Uncertain |
| | 38. | I | 9,0 | 1,0 | Uncertain | 8,0 | 1,0 | Adequate |
| | | F | 5,0 | 2,0 | Uncertain | 5,0 | 1,0 | Uncertain |
| | 39. | I | 9,0 | 1,0 | Adequate | - | - | - |
| | | F | 5,0 | 3,0 | Uncertain | 5,0 | 1,8 | Uncertain |
